# Lived Experiences of Endometriosis‐Related Pain and Perceptions of a Blended Digital CBT Intervention: A Mixed‐Methods Study

**DOI:** 10.1155/prm/6150844

**Published:** 2026-11-06

**Authors:** Suzanne Petersson, Linnéa Engman, Ellen Carlsson, Afra Taygar, Sara Laureen Bartels, Rikard K. Wicksell

**Affiliations:** ^1^ Department of Medicine and Optometry Linnaeus University Kalmar Sweden lnu.se; ^2^ Department of Clinical Neuroscience Karolinska Institute Stockholm Sweden ki.se; ^3^ Region Kalmar County Kalmar Sweden; ^4^ School of Behavioural, Social and Legal Sciences Center for Health and Medical Psychology (CHAMP) Örebro University Örebro Sweden oru.se; ^5^ Pain Clinic Capio Saint Göran′s Hospital Stockholm Sweden; ^6^ Department of Psychiatry and Neuropsychology Mental Health and Neuroscience Research Institute Maastricht University Maastricht the Netherlands maastrichtuniversity.nl

**Keywords:** acceptance and commitment therapy, cognitive-behavioural therapy, endometriosis, internet, pain

## Abstract

**Background:**

Endometriosis affects approximately 10% of women of reproductive age and is associated with reduced health and quality of life yet remains poorly understood and undertreated. The Digital behAvioural HeaLth treatment for chronIc pAin (DAHLIA) aims to enhance behavioural self‐management and resilience to pain through six self‐guided digital modules and regular therapist contact. This study aimed to explore women’s lived experiences of endometriosis‐related pain and their perceptions of the DAHLIA content and structure.

**Methods:**

Six women with endometriosis were consecutively recruited. Semistructured interviews were conducted and analysed using Thematic Analysis. Qualitative findings were complemented by patient‐reported outcome measures collected before and after the treatment and analysed with the Reliable Change Index.

**Results:**

Participants expressed positive views of the treatment, particularly valuing therapist contact via phone/video over in‐person or text‐based communication. A recurring theme was feeling seen and acknowledged, contrasting previous experiences of dismissal in healthcare. Time demands were noted as a challenge. PROMs indicated reductions in pain and mixed outcomes regarding psychological symptoms and pain acceptance, suggesting potential utility of the intervention.

**Conclusion:**

The DAHLIA treatment appears feasible and well‐received, with findings indicating potentially meaningful improvements in physical and psychological domains. This study supports DAHLIA as a promising treatment option for women with endometriosis, warranting further investigation.

**Trial Registration:** ClinicalTrials.gov identifier: NCT05066087

## 1. Introduction

Endometriosis is a chronic, often painful, inflammatory condition that affects approximately 10% of women of reproductive age worldwide, and among women with chronic pelvic pain and fertility issues, the prevalence may be as high as 50% [1]. The condition is characterized by the growth of endometrium‐like tissue outside the uterus, for example, on the ovaries, or in the rectum, bladder or peritoneal cavity [2]. Endometriosis is hormone‐dependent, and its aetiology is believed to involve a combination of hormonal and immune‐related factors [3]. Notably, comorbid health conditions are common among individuals with endometriosis such as irritable bowel syndrome (IBS), overactive bladder and chronic pain [4]. Persistent pain is considered the most severe symptom of endometriosis by those affected [5] and may result from multiple mechanisms, including nociception, inflammation and alterations in pain processing within the peripheral and central nervous systems [6]. Symptoms can be severe even when the endometriotic lesions are small, while larger lesions do not necessarily produce more pronounced symptoms. However, deep infiltrating endometriosis typically results in significant pain [7].

As with other chronic conditions, pain is often accompanied by psychological distress and fatigue, which may further exacerbate the condition and its impact on everyday life [8, 9]. The impact of endometriosis on reproductive and overall health is often severe [3], with research indicating adverse effects on several key health domains, including sexual function [10], psychological well‐being [5] and quality of life [9, 11]. Furthermore, women with endometriosis demonstrate significantly lower salaries, slower salary growth and higher likelihood of leaving the workforce compared to matched controls without endometriosis [12], illustrating the effects of the condition on everyday life as well as important health equity aspects in the society.

Endometriosis is also associated with considerable healthcare costs [13], increased sick days [14] and reduced productivity [14]. Many individuals with endometriosis are misdiagnosed with other physical health (95.1%) and/or mental health problem (49.5%) [15]. A correct diagnosis is often delayed; on average, women with endometriosis receive their diagnosis 7 years following the initial presentation of symptoms [4, 16]. Treatment typically comprises a combination of hormonal therapy and anti‐inflammatory medication, sometimes complemented by surgery [17]. Medical treatments frequently result in side effects and yield variable outcomes across patients [18]. In severe cases, opioids are sometimes prescribed despite the known limitations in effects for chronic pain [19, 20]. This has resulted in four times higher risk of chronic opioid use among women with endometriosis compared to those without the condition [21].

The complexity of this condition necessitates multidisciplinary management comprising medical as well as psychological interventions [9]. Hence, the prevalence and impact of endometriosis call for improved access to effective treatments aimed at increasing resilience and functioning. It is well known that behavioural treatment is critical for optimal and sustainable effects of chronic pain management, with cognitive‐behavioural therapy (CBT) being the most widely utilized and empirically supported approach [22]. Modern forms of CBT, particularly Acceptance and Commitment Therapy (ACT), demonstrate promising results in terms of reducing the impact of chronic pain on daily life, functional disability, depression and quality of life [23]. Although there is yet a scarcity of studies on evidence‐based psychological interventions for people with endometriosis [24], promising effects of CBT on pain and emotional well‐being are emerging [25, 26]. Research on pelvic pain in women with endometriosis suggests that the severity of the pain is associated with pain catastrophizing and avoidant behaviour [27], mechanisms that are central to CBT and ACT.

Importantly, access to effective psychological and behavioural treatments for endometriosis remains limited, with practical barriers such as geographical distance, appointment scheduling, long wait times and restricted clinic hours further hindering availability [28]. Digital treatments are a pathway to increase availability of support [29]. To date, however, research on digital health treatments based on CBT/ACT for endometriosis remains scarce [30]. Furthermore, despite endometriosis negatively affecting a significant proportion of individuals, substantial knowledge gaps remain regarding complex physiological processes [31] as well as individual variations in treatment effects and treatment mechanisms, which prevents the development of effective and accessible treatments, including personalized interventions that match specific patient needs [24, 32]. Since endometriosis typically follows a hormonally regulated, cyclical course, its diagnosis differs from other chronic pain conditions. It is therefore important to evaluate whether and how a treatment developed for general chronic pain may be effective for this specific condition. In this study, participant’s descriptions of suffering, functional limitations and previous experiences of being questioned constitute a contextual frame at the outset of treatment. While several aspects of living with endometriosis have been described previously, the present study adds value by examining these experiences in a population actively seeking digital psychological treatment and by linking lived experiences to engagement with, and responsiveness to, internet‐delivered CBT that includes regular phone/video sessions with a therapist.

## 2. Methods

### 2.1. Aim

To address these knowledge gaps, the aim of this study was twofold: (1) to explore the lived experiences of women actively seeking digital psychological treatment for endometriosis‐related pain and (2) to examine how the Digital behaviourAl HeaLth for chronIc pAin (DAHLIA) treatment—developed for various forms of chronic pain and designed to enhance resilience to distress and pain—was perceived by this population.

### 2.2. Study Design

The present mixed‐methods study used a semistructured interview guide (Appendix A) to explore lived experience of endometriosis and perceived utility of the DAHLIA treatment combined with self‐reported data pre‐ and post‐treatment with Reliable Change Index (RCI) [33] to examine potential treatment effects at the individual‐level.

### 2.3. The DAHLIA Treatment

This study is part of the DAHLIA program, aimed at generating evidence‐based digital health interventions for people with chronic pain [34]. The DAHLIA treatment (Swedish: *Leva med smärta* [English: living with pain]) is grounded in learning theory and builds on an integration of the fear‐avoidance [35] and psychological flexibility models [36]. Thus, the DAHLIA treatment applies behavioural change principles derived from CBT and ACT. The treatment was codesigned in collaboration with patients with chronic pain, clinicians, healthcare stakeholders and pain researchers [34, 37, 38] and is implemented within the existing healthcare infrastructure through the Swedish digital healthcare platform ‘1177 Stöd och behandling’. It targets pain behaviours seen in people with different chronic pain diagnoses, including endometriosis.

The treatment is a guided digital health treatment designed to enhance resilience to pain and distress by improving behavioural self‐management skills, thereby promoting everyday functioning and well‐being. Treatment consists of six modules to be completed over a period of approximately 6–8 weeks. Each module comprises four microsessions (i.e., short section of information), including pain education, reflective prompts and exercises designed to enhance self‐awareness, and assignments to facilitate behavioural change in everyday life (Table A1).

Upon completion of each module, participants meet with their therapist via phone or video, according to patient and therapist preference, in sessions typically lasting around 30 min. The digital platform also enables participants to send secure messages to their therapist, with longer responses typically provided on a weekly basis. Progression through the modules is sequential; the next module is unlocked by the therapist following the completion of the previous module and postmodule communication. Therapists can monitor participant progress in real‐time, including responses to exercises and reflections. If a participant fails to complete sessions as scheduled, therapists can send reminders to support continued engagement. Following completion of the treatment, two booster sessions (phone/video) are scheduled after two and 4 months, respectively. In these booster sessions, the therapist follows up on how the patient maintains and further develops the value‐consistent behavioural changes initiated during digital treatment.

### 2.4. Procedure and Participants

Participants expressed interest in the treatment study by scanning a QR code to access a REDCap page (Research Electronic Data Capture, i.e., a digital tool that supports secure data collection [39, 40], where they received written information about the project, confirmed initial eligibility based on self‐report, signed informed consent form and completed initial self‐assessment questionnaires. Subsequently, a psychologist contacted the participant for assessment based on the study criteria, screened for eligibility (Table 1) and provided additional verbal information about the study and treatment. In this study, participants received the treatment from one of two licenced psychologists (LE and SP), at two different clinics (one in primary care and the other at a specialized pain clinic).

**TABLE 1:** Inclusion and exclusion criteria.

*Inclusion criteria*
• ≥ Age 18 years
• Pain duration ≥ 3 months
• Can communicate in Swedish
• Have access to computer, smartphone, and internet at home
• Able to use email and SMS to receive notifications

*Exclusion criteria*
• Severe psychiatric comorbidity (e.g., suicide risk, psychotic conditions, substance abuse).
• Severe injury/illness requiring immediate treatment or investigation (or expected to worsen within the next 3 months, e.g., cancer treatment, IVF, major surgery).
• Unstable medication (medication started ≤ 2 weeks ago or planned to start within the next 2 weeks).
• Ongoing psychosocial treatment for pain (e.g., CBT or ACT), or completed within the last 3 months.

The sample consists of the first seven participants with endometriosis from a cohort of patients included in the pilot testing of the DAHLIA treatment [38]. Eligible patients were consecutively invited to a user interview. All patients agreed but one could not be reached, and thus, six participants were included in the study. Participants were aged 29–42 years, and most had significant menorrhagia and strong pain since menarche. They were diagnosed with endometriosis between two to 12 years earlier, and the time between first symptoms to diagnosis ranged between four to 24 years. All participants but one were working or studying at the time of the interviews. Five lived with a partner and four had children (Table 2).

**TABLE 2:** Participant characteristics at baseline.

	Participants					
	1	2	3	4	5	6
Age	36	41	35	34	41	28
Occupation	Student	Full‐time	Student	Unemployed	Sick leave	Full‐time
Pain duration (years)	6	4	25	22	12	15
Time since diagnosis (years)	4	2	10	12	5	2.5
Comorbidity	IBS Widespread pain	IBS Migraine fibromyalgia	IBS Headache Widespread pain	Widespread pain	IBS Headache	IBS migraine
Partner (yes/no)	Yes	Yes	Yes	Yes	Yes	Yes
Children (number)	1	0	1	3	2	0

Abbreviation: IBS = irritable bowel syndrome.

## 3. Data Collection

### 3.1. Semistructured Interviews

Semistructured interviews were conducted using an interview guide (Appendix A) and followed a format used successfully in previous studies [41, 42]. Demographic questions regarding age, marital status, duration of endometriosis symptoms, other conditions and treatment experiences were followed by open‐ended questions focused on living with endometriosis, such as ‘How would you describe living with endometriosis?’ and ‘What experiences do you have with healthcare?’. The next set of questions were related to the DAHLIA treatment, including ‘What expectations did you have for the digital treatment?’ and ‘Was there any part of the treatment that you found particularly helpful? Which part?’ and ‘If you could change something about the treatment, what would it be?’. Open‐ended questions were supplemented with general probing questions to elicit richer personal experiences [43]. The interview guide developed for this study was first used in two pilot interviews. As only minor revisions were made to the original guide, data from the first two interviews were included in the analyses. Interviews were conducted via telephone one to three months after treatment completion by a psychologist with no prior relationship with the participants and lasted between 51 and 76 min. Given the coherence of the interviews and their relevance to the study aim, the small sample was considered to hold adequate information power for a Thematic Analysis.

### 3.2. Patient‐Reported Outcome Measures (PROMs)

#### 3.2.1. Pre‐ and Post‐Treatment Assessments

Participants completed PROMs pre‐ and post‐treatment to examine individual‐level treatment outcomes (Table 3). Analyses of the RCI [33] were conducted for the primary and secondary PROMs, except for the Pain Self‐Efficacy Questionnaire (PSEQ‐2), which comprises two items.

**TABLE 3:** Pre‐ and post‐treatment questionnaires.

	Variable	Instrument	*N* items	Total scoring	References
Primary outcome	Pain severity and interference	Brief Pain Inventory—Short Form (BPI‐SF)	4 + 7	0–40 + 0–70	[44–46]
	Self‐efficacy	Pain Self‐efficacy Questionnaire (PSEQ‐2)	2	0–12	[47]
	Pain catastrophizing	Symptom Catastrophizing Scale (SCS)	7	0–14	[48]
	Pain Acceptance	Chronic Pain Acceptance Questionnaire (CPAQ‐8)	8	0–48	[49]

Secondary outcomes	Depression	Patient Health Questionnaire (PHQ‐9)	9	0–27	[50]
	Anxiety	Generalized Anxiety Disorder Assessment (GAD‐7)	7	0–21	[51]
	Perceived stress	Perceived Stress Scale (PSS)	10	0–40	[52]
	Insomnia	Insomnia Severity Index (ISI)	7	0–28	[53]

*Note:* All questionnaires are validated in Swedish.

#### 3.2.2. Treatment Expectancy

At baseline, participants completed questions on treatment expectations: (1) To what extent do you think the treatment offered can remove your pain? (0 = Not at All to 10 = Completely) and (2) How confident are you that this treatment will improve your pain condition? (0 = Not at All to 10 = Fully Confident).

#### 3.2.3. Self‐Reported Improvement and Treatment Satisfaction

At postassessment, participants reported perceived improvements in well‐being, functioning and overall quality of life, using a 6‐point scale ranging from 1 (Deterioration) to 6 (Complete Recovery). These ratings were based on the questions: ‘To what extent do you experience that your well‐being/level of function/general quality of life has improved after taking part in this treatment’ and ‘Rate on a scale from 0 to 10 how satisfied you are overall with the treatment you have received’. Participants rated their global treatment satisfaction on a scale from 0 (Completely Dissatisfied) to 10 (Fully Satisfied).

#### 3.2.4. The Negative Effects Questionnaire (NEQ)

To assess potential adverse events, the NEQ was administered post‐treatment. The NEQ contains 32 items that are scored on a five‐point Likert‐scale (0–4) and differentiates between negative effects attributed to treatment and other factors, plus one open‐ended question inviting respondents to report any other negative incidents or effects not covered by the preceding items [54].

### 3.3. Thematic Analysis

Interviews were transcribed verbatim and analysed using reflexive Thematic Analysis [55] by the interviewer (EC) and a psychologist affiliated to the research group (SP). Thematic Analysis is an experiential and theoretically flexible method compatible with various paradigms within psychology. The primary objective of this analysis is to capture information that is significant in relation to the overall research question, and themes are therefore allowed to vary in size regarding item content. The interviews were analysed as follows:

1. EC and SP individually read and re‐read all interviews to familiarize themselves with the content of the transcripts.
2. Preliminary codes were assigned by SP to describe the content.
3. The codes were reanalysed by EC and SP, and new initial themes for the codes across the dataset were developed.
4. Themes were iteratively developed and reviewed by EC and SP to capture meaningful patterns in relation to the study aim. Codes from subsequent interviews were examined considering the initial thematic framework and either integrated deductively into existing themes or noted as provisional outliers. These outliers were subsequently reconsidered and, where appropriate, inductively informed the development of new themes.
5. All themes were reviewed independently by SP and EC in relation to the codes and interview texts and discussed until consensus was achieved. This process reduced the number of themes to the final set, and some themes were relabelled. When the set of themes was found to be consistent with the data, it was regarded as final.
6. Examples of quotations, representative of the key themes, were selected by SP and EC to be included in the Results section.

In line with the principle of information power, data from the interviews were considered sufficient once the analysis provided a rich and coherent account of the research question, with themes that were well supported across participants. At this stage, additional interviews were not expected to yield substantially new insights, but rather to reinforce the existing thematic patterns.

#### 3.3.1. Methodology and Author Reflexivity

Braun and Clarke’s reflexive Thematic Analysis [55] is well suited for analysing interviews with women living with endometriosis, as the approach acknowledges the inherently interpretative nature of meaning‐making in contexts characterized by complexity, heterogeneity and embodied experiences. Reflexive Thematic Analysis enables a nuanced exploration of how participants make sense of pain, uncertainty and disruptions to identity and everyday functioning—domains that are inherently subjective and shaped by social, relational and healthcare contexts. The analytic team, EC and SP, brought complementary professional backgrounds and perspectives to the reflexive Thematic Analysis. None of the researchers had personal experience of endometriosis, which reduced the risk of over‐identification with participants’ accounts while making reflexive attention to gendered pain discourses particularly important. EC (26 years at the time of analysis) was newly licenced as a psychologist and worked clinically with patients presenting a range of psychiatric conditions. Her perspective contributed contemporary clinical insights and attentiveness to psychological processes relevant to digital interventions. SP (60 years at the time of analysis), mother of a young adult daughter, has more than 3 decades of clinical experience with chronic pain patients, though not in the areas of pelvic pain or endometriosis. Her research focuses on women’s health, including eating disorders, prolonged grief and pain, providing a long‐standing expertise in gendered health disparities and chronic pain trajectories. Together, these diverse backgrounds informed the interpretative process and supported a reflexive, contextually grounded analysis of women’s experiences of endometriosis and the digital CBT‐based intervention. To minimize potential bias, SP who was involved in treatment delivery did not conduct the interviews. The interviews, transcriptions and the qualitative analysis were led by EC, who had no clinical relationship with the participants. SP and EC engaged in ongoing reflexive dialogue to ensure that the interpretations were not shaped by SP’s previous interactions with the participants.

### 3.4. Pre‐ and Post‐PROMs

Individual‐level raw scores were used to describe treatment expectations at baseline, improvement and treatment satisfaction after completed treatment, and negative effect. The limited sample size precluded meaningful group‐level inferences.

#### 3.4.1. RCI

The RCI [33] was calculated as

(1) >RCI=X2−X1SEdiff>,

where *X*_1_ is the pretreatment score, *X*_2_ is the post‐treatment score, and SE_diff_ is the standard error of the difference.

The standard error of measurement (SEM) was computed as

(2) >SEM=SD×1−α>.

The standard error of the difference was computed as

(3) >SEdiff=2×SEM2>.

## 4. Results

### 4.1. Lived Experience of Women With Endometriosis and Pain

Based on the Thematic Analyses, data were organized into four themes and six subthemes (Figure 1).

**FIGURE 1:**
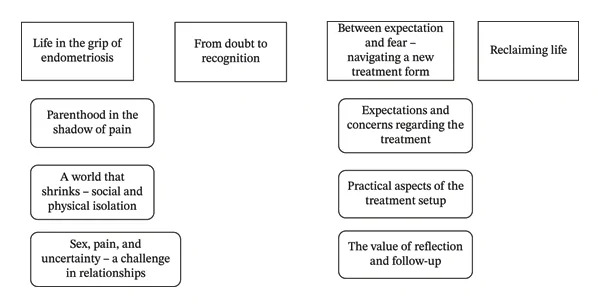
Themes and subthemes.

### 4.2. Life in the Grip of Endometriosis

#### 4.2.1. Parenthood in the Shadow of Pain

Participants described how substantial parts of their lives had been affected by endometriosis. Aspects related to children and parenthood were particularly impacted; every stage in the process—from getting pregnant to managing the parental role—was associated with anxiety and sorrow. There was uncertainty regarding the likelihood of getting pregnant. Other participants expressed a desire for another child, but also profound concern about how another pregnancy might exacerbate symptoms. They reported that pain interfered with being an active parent, such as lifting and carrying their child, which was a source of sadness. Participants described how their children had perceived them as unable to engage in typical parental activities during episodes of severe pain and had therefore become more attuned to their mothers’ well‐being. Parent participants expressed concerns that their children might experience distress as a result of these situations.

*‘My biggest fear was that our son would have to see his mom lying on the floor. Sadly, he has ended up both seeing and hearing when his mother is in pain. He learns from it—he is very considerate and empathetic. But this was not quite the way I wanted it to turn out’.* (Participant 3)

*‘You feel like you can′t manage being a parent and taking care of your home because you’re in pain. You almost end up in a sort of paralysis, like if I get up now, I′ll just collapse into a heap*’. (Participant 4)

*A World That Shrinks—Social and Physical Isolation*

Several participants described feeling ‘trapped’ in their homes and increasingly socially isolated, largely due to the fear of planning activities. Pain and a lack of energy also affected their ability to manage household tasks, leading to feelings of guilt about not being able to cook or clean. In some cases, these responsibilities were entirely taken over by their partner. Travelling was described as challenging, particularly because time spent in a car or a bus exacerbated pain, and overnight stays posed additional difficulties due to pain and sleep disturbances. Participants also frequently perceived themselves as uninteresting or boring in social contexts.

*‘You miss out on a lot with friends and colleagues.* […] *When I wasn’t in the hospital but still in pain, there were times I chose not to do certain things. I chose not to go out for dinner because I knew that if I ate certain foods or sat too long in tight trousers, I’d be in pain. And then—I don’t want to be in pain, so I just cancel it completely. You become a bit isolated that way*’. (Participant 6)

#### 4.2.2. Sex, Pain and Uncertainty—A Challenge in Relationships

Participants described how endometriosis interfered with intimacy. Some participants reported difficulties finding suitable contraceptives, and heavy bleeding posed an additional barrier to sexual activity. All participants experienced pain during and/or after intercourse, and some avoided sexual activities altogether for this reason.

*‘When I am in such pain, I can’t have sex. And you don’t really feel like having sex when you’re tired, exhausted and in pain.* […] *if I’ve had intercourse, I’ve had very deep pain. And it doesn’t go away in an hour, it can stay for several days. So, then it’s also been—well, like this: “no, I don′t want to have sex.” It hurts a week afterwards, too’*. (Participant 5)

A couple of participants described how this had developed into an aversion to all forms of physical contact, such as hugs, due to the fear of experiencing increased pain.

*‘If someone hugs me, I kind of tense up and go into “defence mode.*”’ (Participant 4)

The difficulties with intimacy and sex also affected participants’ partners. One participant described how, although grieving the fact that their intimate life was not as they had hoped, they managed to navigate this together, strengthening their relationship.

*‘I think what he was most upset about was our level of intimacy* […]*, it affected him more. He didn′t feel my symptoms and didn’t really understand why it wouldn’t work* […]*. But of course, his desire was still there even though mine was very absent. But even then, we dealt with it together and did what we could*’*.* (Participant 1)

### 4.3. From Doubt to Recognition

Being diagnosed with endometriosis was generally a prolonged and unclear process, often involving inconsistent information from different physicians and sometimes even from the same physician. Participants described how the determination of whether they had endometriosis frequently shifted, illustrated by one participant who was referred from a regional hospital to a specialist clinic in a larger city and subsequently received two entirely conflicting diagnoses. She later underwent a laparoscopy, only to receive two additional contradictive diagnoses.

*‘I had an MRI that showed endometriosis, but then it wasn’t endometriosis* […]*. And then I got papers after the surgery saying, ‘No, you don′t have endometriosis,’ and then another paper came saying, ‘Yes, you do have endometriosis* […]*. So, it’s been a lot. I felt like a pinball going back and forth for a while*’. (Participant 1)

Participants reported distress related to not being believed and years of dismissive or even demeaning encounters with healthcare professionals, including comments that their symptoms were ‘*just psychological*’, that they ‘*probably aren’t in that much pain*’, that they ‘*only want addictive medication*’ or that they are ‘*overly sensitive*’. These experiences had enduring consequences, undermining participants’ trust in the healthcare system.

*‘Sometimes you almost feel ashamed for seeking help for it*’. (Participant 2)

*‘I’ve also been called a morphine addict, that I’m drug dependent. That I asked for it because I’m screaming and crying from pain, because it hurts so damn much. The doctor asks if I’m experiencing withdrawal when I’m really just in pain. So, it makes you feel very small*’. (Participant 6)

Participants emphasized the importance of feeling acknowledged and heard, particularly given their prior experiences of being dismissed or overlooked. However, several participants described feeling questioned outside healthcare settings as well, by people around them attributing their pain to social circumstances rather than recognizing the medical condition. One participant described frequently staying home from school during childhood due to severe menstrual pain; teachers and the school health team interpreted her absences as signs of bullying or social exclusion, despite her having friends and enjoying school. Other participants reported that employers had speculated that they were being abused by their partner or were experiencing difficulties, assuming this to be the real reason for their frequent sick days. Some participants had changed jobs because workplaces were unable to accommodate their functional limitations or sickness absences or due to the lack of understanding from managers and colleagues:

*‘I had just started the new job when these problems escalated so much. So, I became ‘the one who never showed up.’ I became ‘the one’ they didn’t know if she would come to work or how it would be or if I had to go home or was stuck in the bathroom. And it affected my self-esteem enormously, that I became unreliable, the unreliable colleague* […]*. I want to work but never be ‘that one’ again, ever’*. (Participant 1)

All participants described being unable to plan activities in advance. Also, they described poor self‐esteem due to their inability to meet expectations at work, within their family or among friends. Furthermore, they described a sense of uncertainty about whether they could rely on their own bodies.

*‘It feels tough that you can’t just be stable in some way. And you don’t know when it* (the pain) *will just hit either, you know. That’s kind of the… hard part about it…’.* (Participant 5)

However, most participants described a significant improvement in the way they are treated within the healthcare system today as compared to before, even if still occasionally encountering scepticism. Several participants reported that they now have a clear care plan that helps healthcare staff allocate the right healthcare resources when needed without unnecessary questioning. Generally, the level of knowledge about endometriosis among healthcare providers is also described as significantly higher today. The participants emphasized the importance of respectful treatment from the healthcare, taking patients with endometriosis seriously, involving the patient in their treatment and providing understandable explanations for various decisions.

*‘I especially notice the change when I seek healthcare. That I’ve finally reached the point where they take me seriously when I come in (to the hospital), that I have a care plan. That I don’t have to fight every time I need help.* […] *I don’t have to call the 24/7 medical advice hotline and cry on the phone and despite this being snapped at. Well, it feels like the treatment part (is better) and that must be because they are educating more, I think, and talking more about it*’. (Participant 3)

### 4.4. Between Expectation and Fear—Navigating a New Treatment Form

#### 4.4.1. Expectations and Concerns Regarding the Treatment

Participants reported positive expectations regarding the DAHLIA treatment and the research project. One participant, who had a daughter, expressed a desire to contribute to research aimed at improving healthcare for future generations. Participants described entering the treatment with openness, seeking new perspectives on their situation and tools to better manage life with pain. One participant mentioned feeling desperate to find a treatment that would be effective after numerous healthcare contacts with insufficient results. For some, their positive attitude towards the treatment was influenced by a current, supportive relationship with the healthcare provider who recommended the DAHLIA treatment. In contrast, others reported that previous negative experiences with healthcare generated concerns that the treatment might include judgemental elements or be based on the notion that the participant ‘*just needs to learn to ignore the pain’*.

*‘I thought it would be more like: ‘learn to ignore the pain’* […] *‘it’s not dangerous, just forget about it.’ I thought it would be admonishing and judgmental if you took pain relief sometimes*’. (Participant 1)

*‘I’ve been very open to all the help I can get, from all possible sources: ‘Yes, I’ll take it, I’ll take it.’ Anything just to… be able to get better, feel better, manage emotions and pain better. So yes, I’ve been positive and thought that this might provide me with more tools to manage the pain and anxiety, and to view my behaviour and the patterns I need to work on in a clearer, more structured way’.* (Participant 6)

Participants appreciated the flexibility of being able to undergo the treatment at their convenience and that the treatment was always accessible, which enhanced the reflection process by allowing them to revisit and reflect on previous tasks. The treatment provided guidance and support that participants could continue to utilize independently. Some participants continued the writing exercises on their own after the treatment period, which they experienced beneficial for their progress. Not having to travel to a therapist was also appreciated.

*‘Some questions needed more reflections, so I had to save them and come back to them later to continue writing. And some things I knew right away how to answer. But it was also good that I could go back* [in the program]*. I think that was good. That it was accessible*’. (Participant 2)

*‘It was quite nice that you didn′t have to go to a physical place but could have the contact over the phone or however you wanted, via video calls or in that way. And for me, it was the phone—it worked great*’. (Participant 2)

#### 4.4.2. Practical Aspects of the Treatment Setup

Participants also reported some negative aspects, primarily related to technical issues. One participant mentioned difficulties navigating certain parts of the treatment, noting that substantial scrolling was required to move between content in previous modules and the current one. Some participants described the treatment as very time‐consuming. A few noted that there were repetitions in the middle modules and expressed a preference for the content presented at the beginning and end of the treatment.

*‘It … felt a bit repetitive in the middle when you had to fill in how you thought about all the different parts of life. There, I felt that… a lot of it was pretty much the same for me. The actions and thoughts were very similar*’. (Participant 3)

One participant wished for more physical exercises like the breathing exercise included in the programme. Most participants felt it was positive to write and formulate their thoughts in peace and quiet, but one found it somewhat difficult to put their thoughts into words and felt there was a lot to write. None of the participants preferred a chat function with only written feedback.

*‘(Only chat) wouldn’t suit me. I thought there was enough writing already*’. (Participant 1)

Timing was mentioned as an important component for undergoing the treatment. Participants felt that it was crucial not to have too much going on in their lives during the treatment period but also that the timing within the pain process itself could be decisive for receptiveness to the treatment. On the one hand, it was described as important not to start the treatment too early in the process; on the other hand, some participants expressed that they wished this treatment had been available earlier in their pain journey.

*‘…that patients are prepared to spend some time both writing in this program and reflecting a lot. That maybe you shouldn’t have a completely packed schedule while doing this. To get the most out of it, I think*’. (Participant 3)

#### 4.4.3. The Value of Reflection and Follow‐Up

The therapist contacts after each treatment module were perceived as crucial. In conversations with the psychologist, participants received feedback, additional support when needed and validation. Participants described positive interactions with their psychologists, characterized by understanding and support. By discussing their tasks, participants were able to better absorb the content and deepen their reflections. After the conversation with the psychologist, most participants felt more motivated to tackle the next treatment module.

*‘I don’t think I would have gained the same insights on my own, actually. I would have reflected and conducted the exercises, but then felt like, ‘Oh well, that’s probably how it is.’ And then let it go. Now it was more like, what should I say? Discussing things too, and then you come to new insights… during the conversation, you know*’. (Participant 3)

*‘It’s easy to feel that it’s just hard when you don’t have the energy. That it just becomes another burden. But you kind of knew that with the conversations, after each conversation it felt good and then you got more motivated to do the next chapter*’. (Participant 1)

#### 4.4.4. Reclaiming Life

Participants felt that the treatment prompted them to reflect on themselves. The general reflection was that they had eliminated more activities than necessary in their efforts to manage pain. The treatment helped participants to find a better balance in their lives and identify activities that provide energy. These activities varied among participants—some started exercising at the gym, some reconnected with friends, while others spent more time on breathing exercises and mindfulness.

*‘Since I still reflect a lot on my values and goals, I would say that was the most important thing*’. (Participant 1)

*‘It has really eased up so that I dare to do things again. I dare to sleep away from home, I dare to pick up my children, I play with my children and so on, I do things with them, and I prioritize* [personally important] *things. And I’ve tried to get back into exercising and such, to be able to, for example, kitesurf again. I’m not there yet, but yeah’.* (Participant 5)

Other reflections on the treatment process included better prioritization and making the most out of their time. Some participants discovered that the same values may be relevant across multiple areas of life, with the same tasks being important but within different life areas (e.g., health and leisure interests). One participant reported including her child in household activities, which made it take longer time but added quality time to accomplishing some necessary tasks.

*‘I think I’ve become more creative* … *with everyday solutions. What is fun. Combining usefulness with pleasure.* […] *(the child is) involved and then we do something fun afterwards, once we′ve done something useful. So that has also changed. You see that you don′t have to do everything at once but can split it up a bit. And then feel what we… we find a lot of solutions, simply*’. (Participant 1)

Some participants reported experiencing periods of exhaustion, which had resulted in a reduction in meaningful activities. They reported that the treatment had led to an increase in their participation in meaningful activities. Changes in valued activities were associated with reduced isolation, greater energy and enhanced creativity. All participants increased their time spent on leisure activities despite feeling a lack of energy and discovered how this brought back energy and joy. With increased energy and reduced avoidance behaviour, the treatment also reduced the impact of exhaustion symptoms on their lives. Some participants also received feedback from people around them that they seemed to be doing better.

*‘People around me noticed that I have changed, that I seemed more content in some way.* […] [*I have*] *perhaps managed to do things in a completely different way*’. (Participant 2)

*‘I might not have expected it to lead to so much change. And particularly, I was surprised by what changed. I had thought it was more concretely focused on the pain, but it also helped me with my exhaustion problems* […]*. So, it might be important to say that I feel I have received a lot of help in managing my exhaustion even though this CBT is about pain*’. (Participant 5)

## 5. Pre‐ and Post‐PROMs

### 5.1. Time in Treatment and Treatment Expectancy

All participants completed the six sessions included in the treatment programme. However, the total duration of treatment varied between six and 18 weeks (see Table 4). Three participants completed the programme within 6 weeks. Prior to the first module, participants rated the extent to which they believed that the DAHLIA treatment would be a reasonable approach for alleviating pain, as well as their confidence that it would improve their pain condition. Their ratings indicated moderate‐to‐high treatment expectations (5–7 out of 10) but low‐to‐moderate confidence in the treatment (2–5 out of 10).

**TABLE 4:** Treatment specifics and treatment expectations.

Participant	Treatment length (weeks)	Format of contact (number of sessions)	Treatment expectation	Treatment confidence
1	6	Telephone (6)	5	5
2	12	Telephone (2)/chat (4)	6	5
3	6	Telephone (6)	7	2
4	18	Telephone (6)	5	5
5	6	Telephone (6)	5	5
6	12	Telephone (5)/chat (1)	7	5

*Note:* treatment expectation (0–10), treatment confidence (0–10).

### 5.2. Primary Outcome Measures

Table 5 presents pre‐ and post‐treatment scores for the primary outcome measures. One participant did not complete the post‐treatment assessment, resulting in missing data. However, her pretreatment assessment scorings did not deviate from the other participants. Participant 6 demonstrated reliable improvement across all PROMs, whereas Participant 5 showed reliable improvement in all primary PROMS except catastrophizing (measured by the SCS). Participant 2 exhibited minimal change between pre‐ and post‐treatment scores. Four participants reported reduced pain intensity at follow‐up, as measured by the BPS‐S.

**TABLE 5:** Descriptive statistics of primary outcomes pre‐ and post‐treatment.

	Pain intensity (BPI‐S)			Pain interference (BPI‐I)			Pain self‐efficacy (PSEQ)		Catastrophizing (SCS)			Acceptance (CPAQ)		
	Pre	Post	RCI	Pre	Post	RCI	Pre	Post	Pre	Post	RCI	Pre	Post	RCI
1	4.5	1.5	**−5.12**	1.14	0	−0.75	9	10	8	6	−1.91	28	32	0.74
2	7.25	7.25	0	6.86	6	−0.55	9	10	6	6	0	25	24	−0.19
3	6.75	—	—	9.57	—	—	10	—	5	—	—	27	—	—
4	6.75	3.75	**−5.12**	8.71	7.43	−0.84	3	6	7	5	−1.91	15	21	1.12
5	4.75	0	**−8.11**	8.86	0	**−5.84**	2	2	12	10	−1.91	9	22	**2.42**
6	3.75	1.75	**−3.42**	4.57	2.29	**−1.50**	5	9	11	7	**−3.82**	11	28	**3.16**

*Note:* BPI‐S, Severity subscale of the Brief Pain Inventory—Short Form (0–10); BPI‐I, Interference Subscale of the Brief Pain Inventory—Short Form (0–10); PSEQ, Pain Self‐Efficacy Questionnaire (0–6); SCS, Symptom Catastrophizing Scale (0–14); CPAQ, Chronic Pain Acceptance Questionnaire (0–24). RCI = Reliable Change Index, **≥** **1.96** (bold) was considered a reliable change.

### 5.3. Secondary Outcome Measures

As for the primary PROMs, participants exhibited heterogeneous patterns of change on the secondary PROMs. Participant 2 scored in the mild‐to‐moderate range for depression, anxiety and perceived stress at baseline, but reported severe insomnia symptoms, which increased further at the postassessment. Participant 4 showed severe depression and anxiety on the PHQ‐9 and GAD‐7. Her perceived stress was high at baseline and increased further at postassessment, and her insomnia scores also fell within the clinically severe range. In the interview, her narratives aligned with those of the other participants regarding increased acceptance, reduced catastrophizing and engagement in valued activities. However, her overall life situation was markedly strained, as she was caring for three children with psychiatric conditions. Participant 5 demonstrated reliable improvement across all secondary PROMs, whereas Participant 1 showed reliable improvement in depression and insomnia. Sleep outcomes were mixed; two participants reported improved sleep (RCI ≥ 1.96), while two reported deteriorations. Initial anxiety levels, as assessed with the Generalized Anxiety Disorder (GAD‐7) scale, were generally minimal, except for Participant 4 (Table 6).

**TABLE 6:** Descriptive statistics of secondary outcomes pre‐ and post‐treatment.

	Depression (PHQ‐9)			Anxiety (GAD‐7)			Perceived stress (PSS)			Insomnia (ISI)		
	Pre	Post	RCI	Pre	Post	RCI	Pre	Post	RCI	Pre	Post	RCI
1	11	4	**−2.75**	5	2	−1.39	13	6	−1.67	24	18	**−2.87**
2	9	10	0.39	4	3	−0.46	11	7	−0.95	21	25	1.91
3	20	—	—	9	—	—	20	—	—	21	—	—
4	21	19	−0.79	19	21	0.93	29	34	1.19	26	28	0.96
5	18	6	**−4.72**	10	6	**−1.85**	28	12	**−3.81**	18	13	**−2.39**
6	10	8	−0.79	7	6	−0.46	21	17	−0.95	14	11	−1.43

*Note:* PHQ‐9, Patient Health Questionnaire (0–27); GAD‐7, General Anxiety Disorder (0–21); PSS, Perceived Stress Scale (0–40); ISI, Insomnia Severity Index (0–28). RCI= Reliable Change Index, **≥ 1.96** (bold) was considered a reliable change.

#### 5.3.1. Self‐Reported Improvement and Treatment Satisfaction

Four participants rated improvement regarding their overall well‐being, levels of function and quality of life as moderate to high, while also reporting a moderate‐to‐high satisfaction with taking part of the treatment (Table 7).

**TABLE 7:** Self‐reported improvement and treatment satisfaction.

Participant	Well‐being	Function	Quality of life	Treatment satisfaction
1	—	—	—	—
2	5	4	5	10
3	—	—	—	—
4	4	5	5	10
5	5	4	4	7
6	5	5	5	8

*Note:* Self‐reported improvement (1–6). Treatment satisfaction (0–10).

#### 5.3.2. Negative Effects

On the NEQ administered post‐treatment; three participants reported no negative effects; two participants (5 and 6) reported negative effects on factors such as sleep, stress, anxiety and worry caused by other circumstances outside of the treatment (as reported in the open question); and Participant 3 did not complete the assessment. Also, some reported that taking part in the treatment increased negative memories and unpleasant feelings.

## 6. Discussion

This study examined how self‐referred patients with endometriosis experience everyday challenges and evaluated a digital health treatment targeting chronic pain with the aim of supporting resilience, functioning and well‐being. Although the sample size was small, it was broadly comparable to those in previous studies, particularly with respect to experiences of living with endometriosis, comorbidity with IBS and chronic pain [4, 56]. Descriptions of suffering, quality of life and prior experiences of being dismissed provide a contextual frame at treatment entry, together with accounts of a process associated with participant’s experience of the treatment.

Participants’ narratives regarding experiences of living with endometriosis and healthcare experiences largely aligned with previous research [9]. Their descriptions of significant interference in aspects of life, including challenges in fully engaging with their children, have been described in previous studies [57, 58]. Additional challenges encompassed social isolation [58, 59], and difficulties with having sex due to pain or heavy bleeding [2]. Participants also reported negative healthcare experiences, including feelings of distrust, instances of disrespectful treatment and an extended diagnostic process [16, 60, 61]. Furthermore, participants described encountering scepticism and dismissive attitudes from colleagues and employers [60]. Consequently, two participants had pursued retraining to enhance their ability to engage in vocational work, while one was on sick leave during the treatment period. A large register‐based cohort study found that women with endometriosis were less likely to be employed or enrolled in education than women without the condition and also reported increased absenteeism, reduced productivity and diminished work ability [62].

Across both qualitative narratives and quantitative outcomes, participants described the treatment as meaningful, validating and easy to understand. This appraisal aligns with the observed reductions in pain intensity and pain interference, findings that are consistent with previous CBT research [24]. At the same time, the quantitative PROM data revealed more modest and heterogeneous effects than the qualitative reports. This heterogeneity mirrors earlier DAHLIA findings across varied chronic pain diagnoses [63]. Rempert et al. [9] emphasize a substantial heterogeneity in symptom presentation and impact on daily functioning for women with endometriosis. They reported individual differences in how problems in specific domains affect the quality of life, depending on life stage, context and personal priorities, contributing to variation in PROMs, symptom profiles, and treatment needs [9]. This framework may offer a plausible explanation for the heterogeneous outcomes observed in the present study, where participants differed in both baseline burden and the domains most salient to their well‐being. Within the scope of this study and due to the small sample, it is not possible to determine whether these discrepancies reflect random variation or whether a systematic pattern would emerge in a larger sample. Future studies should therefore explore these differences more thoroughly.

Building on CBT and ACT, the DAHLIA treatment (Appendix B) emphasizes values and acceptance alongside exposure and emotion‐regulation strategies, mechanisms theorized to strengthen resilience and adaptive functioning [35, 36]. Both interview data and PROMs indicated increased acceptance, most notably among participants with low baseline acceptance, aligning with their accounts of gaining a stronger sense of capability in managing their condition. Participants described how working with avoidance and engaging in exposure helped them re‐enter valued activities, which in turn contributed to a richer everyday life and increased energy. They reported that reconnecting with meaningful activities reduced isolation, enhanced creativity, and restored positive emotions and life balance. Despite initial fatigue, all participants increased their leisure engagement and discovered that this generated renewed energy and joy. As avoidance decreased and energy improved, the impact of exhaustion symptoms diminished, and several participants noted that people around them perceived them as doing better. The DAHLIA treatment also includes CBT components targeting catastrophizing and avoidant behaviours, mechanisms known to contribute to increased pain interference [27] and depressive symptoms [64]. Yet, among the women in this study, changes in catastrophizing and psychological PROM scores were modest.

Initial concerns that the treatment content might be judgemental or scrutinizing were not borne out; all participants reported positive experiences upon completion. This corresponds with previous findings on fears of not being taken seriously when seeking psychological treatment [65]. Participants emphasized the importance of feeling acknowledged and heard, particularly given their prior experiences of dismissal. The digital format was appreciated for its flexibility, and although face‐to‐face therapy was initially preferred in earlier studies [65], participants in the present study adapted well to the digital delivery. Consistent with research on blended treatment approaches [62], participants valued the phone or video contact between modules. All preferred synchronous communication over text‐based chat, and none expressed a desire for face‐to‐face meetings—a combination previously associated with favourable treatment outcomes [66, 67]. The primary negative experience concerned the time required to engage with the modules, which exceeded expectations.

Although several of the qualitative themes identified in this study have been described previously, the present work offers a contribution by examining these experiences in a population actively seeking digital psychological treatment for endometriosis‐related pain. Through the integration of qualitative accounts with treatment engagement and outcome data, the study extends existing knowledge by clarifying how well‐established challenges reveal possible barriers relevant for digital treatment interventions. Overall, the integration of qualitative and quantitative findings suggests that the digital intervention may hold promise for improving resilience and functioning in women with endometriosis. However, the variability in PROM outcomes and the small sample size underscore the need for adequately powered clinical trials to validate these preliminary results.

## 7. Limitations

Participants in this study generally reported positive experiences with the treatment. However, there are some limitations that warrant consideration. First, although the sample size was adequate for the qualitative analysis, it was insufficient to support meaningful inferences from the PROM data. Second, participation in the study was voluntary, and the participants actively chose to enrol after receiving information from healthcare providers, expressing interest via a QR‐code or online link. All participants completed the intervention, which may imply that they were more open minded and positively predisposed than most patients in healthcare, potentially limiting the generalizability of the findings. Third, in this study, conventional standardized instruments for general chronic pain were employed. It is possible that the results would have been more consistent had instruments specifically targeting endometriosis been used. Fourth, using the RCI has been debated [68]; however, the sample in this study was too small for more robust statistical methods. Finally, endometriosis represents a distinct subgroup within chronic pain populations, and it is plausible that an intervention specifically tailored to individuals with endometriosis might have produced more favourable outcomes. Psychoeducation regarding endometriosis and its multifaceted impacts has been identified as a key therapeutic component in supporting patients’ management of the condition [24]. The DAHLIA treatment provides psychoeducation on general chronic pain, which may not meet this group’s needs. Incorporating more endometriosis‐specific examples and information could have enhanced participants’ sense of recognition and potentially amplified the positive outcomes. Future research aimed to design treatments that match specific needs may, thus, build on findings from the present study. Endometriosis encompasses more than pain alone; it is characterized by a diverse range of symptoms, hormonal fluctuations and cyclical patterns that distinguish it from conditions such as low back pain [24].

## 8. Conclusions

The present study contributes to the rapidly growing field of eHealth by articulating the voices of a largely neglected subgroup of women with endometriosis and chronic pain. The findings indicate that the DAHLIA intervention is feasible, acceptable and perceived as clinically useful by participants. Participants described shifts in fear and avoidance, greater balance and intentional prioritization, and increased engagement in meaningful activities, with some also reporting enhanced energy, creativity and positive affect. Participants described the phone/video sessions as a prerequisite for treatment completion and, together with the brief six‐module format, may have supported full adherence. Given the small sample and substantial variability in PROM outcomes, these self‐reported changes should be interpreted cautiously and cannot be taken as evidence of treatment effectiveness. Instead, the results offer preliminary indications of the potential clinical utility of the DAHLIA intervention. To establish efficacy and clarify mechanisms of change, adequately powered randomized controlled trials are required. Collectively, participants described the experience as a reclamation of life. The findings may contribute to future development of tailored digital interventions and guide clinicians in integrating digital psychological treatment into holistic care for women with the condition.

**Nomenclature**
ACT: Acceptance and Commitment Therapy
BPI‐I: Interference Subscale of the Brief Pain Inventory
BPI‐S: Severity Subscale of the Brief Pain Inventory
BPI‐SF: Brief Pain Inventory—Short Form
CBT: Cognitive‐Behavioural Therapy
CPAQ: Chronic Pain Acceptance Questionnaire
DAHLIA: Digital Behavioural Health Treatment for Chronic Pain
GAD‐7: General Anxiety Disorder (Questionnaire)
IBS: Irritable Bowel Syndrome
iCBT: Internet‐Delivered Cognitive Behavioural Therapy
ISI: Insomnia Severity Index
IVF: In Vitro fertilization
NEQ: Negative Effects Questionnaire
PHQ‐9: Patient Health Questionnaire
PROM(s): Patient‐Reported Outcome Measure(s)
PSEQ: Pain Self‐Efficacy Questionnaire
PSS: Perceived Stress Scale
QR (code): Quick Response (Code)
RCI: Reliable Change Index
REDCap: Research Electronic Data Capture
SCS: Symptom Catastrophizing Scale
SMS: Short Message Service

## Author Contributions

Suzanne Petersson and Linnéa Engman designed the study and drafted the initial manuscript. Ellen Carlsson conducted the interviews and contributed to the interpretation and processing of the interview data together with Suzanne Petersson. Afra Taygar and Linnéa Engman prepared the PROM data. Rikard K. Wicksell served as the principal investigator and leader of the DAHLIA research group and contributed to revising the manuscript. Sara Laureen Bartels acted as an advisor and sounding board and contributed to manuscript revisions. All authors discussed and commented on the manuscript.

## Funding

The Research Section of Region Kalmar County funded time for interviews and interview transcriptions (2024: RK‐1 012 747).

## Disclosure

All authors approved the final version.

## Ethics Statement

The study was conducted in accordance with the Helsinki Declaration and received approval from the Ethics Review Authority (Reference Nos.: 2021–02437; 2023‐03319‐02; and 2024‐03857‐02). [2023‐10‐03]. Data were handled confidentially, ensuring that no statements would be traced back to participants.

## Consent

Participants in the study were informed and signed a consent document. The document was approved by the Ethics Review Authority (Reference Nos.: 2021–02437; 2023‐03319‐02; and 2024‐03857‐02).

## Conflicts of Interest

Suzanne Petersson and Linnéa Engman provided treatment but were not involved in the interviews. The other authors declare no conflicts of interest.

## Data Availability

The data that support the findings of this study are available from the corresponding author upon reasonable request.

## Appendix A Interview Guide: Experiences of Living with Endometriosis and the Internet‐Based Cognitive‐Behavioural Therapy (iCBT) Program *Living with Pain*

Purpose
To explore the lived experience of endometriosis and qualitatively evaluate the iCBT program *Living with Pain* for women diagnosed with the condition.
Research Question
How do women with endometriosis experience their symptoms and overall situation, and how does the iCBT program *Living with Pain* work for this group?
Introduction
Initial conversation outlining the scope of the interview and obtaining verbal informed consent.
Demographic questions
• Age
• Marital status/partner
• Number of years living with pain or other symptoms related to endometriosis
• Time since diagnosis of endometriosis
• Experience of other types of pain and duration
• Any comorbid conditions
Experiences of endometriosis
• Do you experience more discomfort from endometriosis or other types of pain? (clarify if it’s a mix)
• How would you describe what it’s like to live with endometriosis?
• Has endometriosis affected your life? In what ways?
• If so: In what ways does endometriosis limit or hinder you?
• Relationships? Sexuality? Social life? Thoughts about the future/anxieties?
• What helps you when things feel difficult? (Use the participant’s own wording for distress if applicable)
• When do you feel at your best?
• Previous healthcare contacts (related to endometriosis)
• What kind of support or interventions have you received from healthcare services?
• What has your experience with healthcare been like?
• Is there anything you wish you had received more or less of from healthcare providers? *(Capture aspects such as treatment, attitudes, and provider knowledge)*
• Experiences of *living with pain*
• What expectations (positive or negative) did you have before starting *Living with Pain*? Have these changed over time?
• What are your thoughts on the treatment? Has it led to any changes for you?
• Was there any part of the treatment that you found particularly helpful?
• Is there anything you felt was missing, and if so, what?
• If you had the opportunity to change something about the treatment, what would you change?
• How did you experience the setup of having contact with a therapist alongside the digital treatment?
• In what ways do you think the treatment would have been different without that option?
• Closing
• Repeat the study’s purpose: *To explore the experience of living with endometriosis and evaluate the iCBT program Living with Pain for individuals with endometriosis and/or chronic pain.* Then ask: *Is there anything we haven’t talked about that you feel is important to bring up?*
• Of everything we’ve discussed during the interview—*what do you think I should especially take with me?*

## Appendix B

**TABLE A1:** Overview of treatment content.

Module	Session	Content of the microsessions
1—You and your pain	1	Psychoeducation on recurring pain and psychological treatment of pain
	2	Psychoeducation on the interplay between psychological factors and pain
	3	The role of behaviour in connection with pain and vicious circles
	4	Functional analysis of behaviour in relation to long‐ and short‐term consequences

2—Where do you want to go?	1	Introduction to values in different areas of life
	2	Life values in relation to family and close relationships
	3	Values in relation to friends and health
	4	Values in relation to work/education and leisure activities

3—What do you want to achieve?	1	Values in relation to goals and goals in relation to family and close relationships
	2	Goals in relation to friends and health
	3	Goals in relation to work/education and leisure activities
	4	Reflections on a life led in the direction of one’s values

4—moving forward	1	Taking steps towards goals based on values
	2	Taking difficult steps towards goals even when it is difficult
	3	Negative thoughts and emotions as part of change and strategies to handle catastrophic thoughts
	4	Using the support from others

5—increasing strength and resilience	1	Building skills to handle difficult change—i.e., decreasing avoidance behaviour through exposure
	2	Dissecting goals and steps into manageable parts
	3	Emotion‐regulation strategies for strong affects
	4	Acceptance and validating strategies for self‐criticism

6—the road ahead	1	Creating detailed plans of steps to take in relation to goals
	2	Repeating actions to form new habits
	3	Setting up plan for maintaining progress after treatment
	4	Reflections on treatment progress and planning for the future

*Note:* Ref.[37].
